# The LncRNA FEZF1-AS1 promotes tumor proliferation in colon cancer by regulating the mitochondrial protein PCK2

**DOI:** 10.32604/or.2022.03553

**Published:** 2022-08-01

**Authors:** HUAMIN WANG, YANTING WU, ZHENLEI WANG, YUHANG CHEN, JINYU MO, WEN GUAN, YALI ZHANG, HONGLIANG YAO

**Affiliations:** 1 Guangdong Key Laboratory of Animal Conservation and Resource Utilization, Guangdong Public Laboratory of Wild Animal Conservation and Utilization, Institute of Zoology, Guangdong Academy of Sciences, Guangzhou, 510260, China; 2 Department of general surgery, The Affiliated Cancer Hospital of Zhengzhou University, Henan Cancer Hospital, Zhengzhou, 450008, China

**Keywords:** Long non-coding RNA (LncRNA), Colon cancer, Phosphoenolpyruvate carboxykinase2 (PCK2), Tricarboxylic acid cycle (TCA), Glycolysis, Oxidative phosphorylation (OXPHOS)

## Abstract

LncRNAs and metabolism represents two factors involved in cancer initiation and progression. However, the interaction between lncRNAs and metabolism remains to be fully explored. In this study, lncRNA FEZF1-AS1 (FEZF1-AS1) was found upregulated in colon cancer after screening all the lncRNAs of colon cancer tissues deposited in TCGA, the result of which was further confirmed by RNAscope staining on a colon tissue chip. The results obtained using FEZF1-AS1 knockout colon cancer cells (SW480 KO and HCT-116 KO) constructed using CRISPR/Cas9 system confirmed the proliferation, invasion, and migration-promoting function of FEZF1-AS1 *in vitro*. Mechanistically, FEZF1-AS1 associated with the mitochondrial protein phosphoenolpyruvate carboxykinase (PCK2), which plays an essential role in regulating energy metabolism in the mitochondria. Knockdown of FEZF1-AS1 greatly decreased PCK2 protein levels, broke the homeostasis of energy metabolism in the mitochondria, and inhibited proliferation, invasion, and migration of SW480 and HCT-116 cells. PCK2 overexpression in FEZF1-AS1 knockout cells partially rescued the tumor inhibitory effect on colon cancer cells both *in vitro* and *in vivo*. Moreover, PCK2 overexpression specifically rescued the abnormal accumulation of Flavin mononucleotide (FMN) and succinate, both of which play an important role in oxidative phosphorylation (OXPHOS). Overall, these results indicate that FEZF1-AS1 is an oncogene through regulating energy metabolism of the cell. This research reveals a new mechanism for lncRNAs to regulate colon cancer and provides a potential target for colon cancer diagnosis and treatment.

## Background

Colorectal cancer (CRC) is one of the leading causes of cancer-related deaths worldwide [1]. Initiation and progression of CRC involves gene mutation, genome instability, abnormalities in DNA or histone modification, and ncRNA dysregulation. Thereinto, long non-coding RNAs (lncRNAs) have garnered increasing attention over the last decade [2–4]. LncRNAs are transcripts over 200 nucleotides that are not translated into proteins and can be classified into intergenic transcripts, enhancer RNAs, and sense or antisense transcripts corresponding to positions of protein-coding genes [5]. It has been reported that lncRNAs function in cancers using different mechanisms, including transcriptional regulation in *cis* or *trans*, interaction with microRNAs or proteins, and organization of nuclear domains [6–8]. However, far more work is required to fully discover the biological functions of the vast majority of lncRNAs.

FEZ family Zinc Finger 1-Antisense RNA1 (FEZF1-AS1) is a newly discovered lncRNA, with a length of 2653bp. FEZF1-AS1 is located on chromosome 7q31.32, contains three splicing variants and the full-length cDNA was first discovered in 2004 [9]. Until 2016, FEZF1-AS1 was first reported to be up-regulated in human primary CRC and was associated with colorectal cancer (CRC) metastasis and poor prognosis [10]. After then, FEZF1-AS1 was successively found to be up-regulated in kinds of malignancies, like pancreatic cancer (PC), hepatocellular carcinoma (HCC), breast cancer (BC), non-small-cell lung cancer (NSCLC) and gastric cancer (GC) [11–15]. Meanwhile, mechanisms underlying FEZF1-AS1 in cancers were uncovered. At transcriptional level, FEZF1-AS1 accelerates gastric progression by inhibiting transcription of P21 via recruiting histone modification proteins [15]. Similar is, FEZF1-AS1 decreases levels of P57 and E-cadherin in LAD (Lung adenocarcinoma) or NSCLC (Non-small-cell lung cancer) cells by recruiting RNA binding proteins EZH2 and LSD1 into their promoter regions, promoting malignant progression of cancers [14,16]. Other than these, FEZF1-AS1 serves as a miRNA sponge to undirectly regulate protein levels. For example, FEZF1-AS1 binds to miR-610, miR-30a, miR-107 as ceRNA to up-regulate AKT3, nanog and FEZF1, respectively [17–19]. No matter what manner, dysregulation of FEZF1-AS1 integrate some pathways like EMT, Wnt/β-catenin and PKM2/STAT3 to facilitate cancer development [20]. However, the function and mechanism of FEZF1-AS1 need more studies to be fully clarified.

Metabolic reprogramming is now being considered hallmark of cancer [21]. In the 1920s, cancer cells were found to convert glucose into lactate even with sufficient oxygen (the “Warburg effect”), which gave birth to cancer metabolism research [22]. Subsequently, various cancers were shown to increase glucose uptake for aerobic glycolysis, the caron containing intermediates of which are used for the synthesis of non-essential amino acids, nucleotides, and lipids [23–26]. Compared with glycolysis, the tricarboxylic acid (TCA) cycle, a central hub for energy metabolism, biomass precursor production, and redox balance, was recently found to be closely related to cancers [26,27]. Loss or misfunction of enzymes of TCA cycle results in a broad spectrum of cancers. IDH1, converting isocitrate to α-ketoglutarate (α-KG), was mutational in secondary glioblastoma and acute myeloid leukemia [28,29]. Succinate dehydrogenase (SDH), catalyzing the oxidation of succinate to fumarate in the TCA cycle, has been reported to promote hereditary paragangliomas and pheochromocytomas [30–35]. It was found that tumor cells uncouple glycolysis from TCA to oxidate glucose via aerobic glycolysis, and then anaplerosis was upregulated to replenish the TCA cycle intermediates [26,36]. Two reactions have been shown to fulfill anaplerosis: the first is glutaminolysis. Glutamine is considered another critical nutrient, other than glucose, for proliferating cancer cells and enables the central role of the TCA cycle [37,38]. Glutaminolysis converts glutamine to α-ketoglutarate in a two-step reaction and plays significant roles in Myc-driven cancers [39,40]. The second is pyruvate carboxylation catalyzed by pyruvic carboxylase (PC), transforming pyruvate to oxaloacetate. Non-small cell lung cancer (NSCLC) cells use PC as the primary anaplerotic reaction to support growth, while glutaminolysis only plays a supportive role [41,42]. Up-regulation of PC protein levels supports breast cancer cell survival when there is glutamine deprivation [43]. Other than these factors, OXPHOS was recently emerging as potential target in tumor therapy for its upregulation in leukemias, pancreatic ductal adenocarcinoma and other high OXPHOS subtype melanoma. However, drugs for OXPHOS should be carefully manipulated because there are cancers with downregulated OXPHOS [44,45]. All in all, mentioned above together with abnormal metabolism of amino acids, fatty acids are actively involving in cancer initiation and progression with known or unknown mechanisms.

The metabolic pathways in cells are not self-existent but mutually connected. Mitochondrial protein phosphoenolpyruvate carboxykinase (PCK2) was one of the central connectors of glycolysis, TCA cycle, and gluconeogenesis, catalyzing mitochondrial oxaloacetate (OAA) to phosphoenolpyruvate (PEP), which is the second step of gluconeogenesis. Concerning TCA cycle, on one hand, PCK2 plays a vital role via increasing efflux of metabolic intermediates. Non-small-cell lung cancer cells (NSCLC) with PCK2 depletion show decreased levels of pyruvate and citrate from glutamine [46]. On the other hand, PCK2 participates in pyruvate cycling in glucose-starved cells by regenerating pyruvate from OAA to feed acetyl-CoA for the citrate synthase in TCA cycle [46,47]. PCK2 also was shown to participate in OXPHOS in tumor repopulating cells [48] and dysregulation of PCK2 was found in many cancer types [49,50].

Metabolic pathways are more like a sophisticated network that must be accurately regulated. We wondered whether there was an association between lncRNAs and the metabolic pathway. In this study, we demonstrated that FEZF1-AS1 is upregulated in colon cancers. RNA pull-down plus mass spectrometry demonstrated FEZF1-AS1 interacts with PCK2 *in vitro*. Knockout and rescue experiments indicated that FEZF1-AS1 inhibited colon cancer cell proliferation, invasion, and migration, partially dependent on PCK2, by disturbing the homeostasis of energy metabolism of the mitochondria.

## Materials and Methods

### Analysis of TCGA data

High-throughput sequencing FPKM data of TCGA COAD (colon cancer) and prognosis data of related patients, including 41 normal tissues and 453 tumor tissues, were extracted. Among the patients, there were 430 patients with survival information. Differential gene analysis was acquired using the linear fitting method (limma), and function log(express_rec,2) was used for normalization. Among all the differentially expressed genes, the upregulated lncRNAs were listed separately, of which the most top 10 were picked. According to the expression of FEZF1-AS1, patients were divided into high expression group and low expression group according to the median, and a KM curve was drawn. Log-rank was used to analyze the difference between the two groups, and the significance was marked on the graph.

### RNAscope

The studies involving human participants were reviewed and approved by the insititutional review board of Shanghai Outdo Biotechnology Co., Ltd. (China). All patients provided their written informed consent to participate in this study.

Tissue microarray chips containing 93 colon cancer tissues and 83 paired adjacent normal colon tissues and the associated clinicopathological information were purchased from Shanghai Outdo Biotech Co., Ltd. (Shanghai, China). Clinicopathological information of these patients is listed in Table 1.

**Table 1:** Correlation between FEZF1-AS1 expression of cancer and clinicopathological characteristics

	Variables	FEZF1-AS1 expression		Total	χ^2^	*p* value
		Low	High			
Age (year)					0.974	0.324
	≤65	23	25	48		
	>65	17	28	45		
Sex					1.361	0.243
	Female	17	29	46		
	Male	23	24	47		
Size					3.012	0.083
	≤5 cm	16	31	47		
	>5 cm	21	19	40		
Grade					4.083	0.043
	I/II	30	48	78		
	III	10	5	15		
T stage					1.261	0.262
	T1, T2	3	8	11		
	T3, T4	37	45	82		
N stage					1.068	0.301
	N0	23	36	59		
	N1, N2	17	17	34		
M stage					0.020	0.889
	M0	38	50	88		
	M1	2	3	5		
TNM stage					1.171	0.279
	I/II	22	35	57		
	III/IV	18	18	36		

The RNAscope probe targeting FEZF1-AS1 was designed and synthesized by the Advanced Cell Diagnostics company (ACD, Newark, CA, USA). Detection of the FEZF1-AS1 expression was performed on the colon tissue array using an RNA-scope 2.5 high definition-brown assay kit according to the manufacturer’s instructions (ACD, Newark, CA, USA). In brief, after dewaxing, ProteaseK (5 μg/mL) digestion of samples was performed for 2 min at 37°C followed by rinsing the samples two times in 0.01% PBT (0.01% Tween-20 in PBS, pH 7.4). Target probe hybridization (hybridization buffer 1: 6 × SSC (1 × SSC is 0.15 mol/L NaCl, 0.015 mol/L Na-citrate), 25% formamide, 0.2% lithium dodecyl sulfate, blocking reagents) was performed at 50°C for 1 h. Then samples were washed using 5 × SSC (50°C), 1 × SSC (50°C), 0.2 × SSC (50°C), 0.2 × SSC (RT) and 1 × PBS (RT), each for 5 min. For detection, anti-DIG-AP Fab fragments were added to samples and incubated overnight at 4°C. The next day, NBT/BCIP was performed in dark at 30°C for h to make the positive point to be visualized. Prior to imaging, samples were rinsed in 0.01% PBT, three times and 5 min each time, nuclear fast red for 10 s and mounted in resinene. The images were acquired with an aperio ePathology scanner (Leica, Wetzlar, Germany). Using the images, signals were scored based on the average number of dots per cell using criteria as follows: 0 (no staining or <1 dot/10 cells), score 1 (1–2 dots/cell), score 2 (2–3 dots/cell, no or very few dot clusters), score 3 (4–6 dots/cell, less than 10% positive cells have dot clusters), score 4 (>6 dots/cell, more than 10% positive cells have dot clusters).

### Cell culture

CaCo_2_ and SW480 were purchased from Hunan Fenghui Biotechnology Co., Ltd. (China). LoVo and T84 were purchased from GuYan Biotech Co., Ltd. (Shanghai, China). HCT-116 was purchased from procell Life Science&Technology Co., Ltd. (Wuhan, China). All cells were authenticated by str. CaCo_2_ cells were cultured in McCoy’s 5A medium (Sangon Biotech Co., Ltd, Guagnzhou, China) with 10% Fetal bovine serum (FBS) (Gibco, Wubo biotech Co., Ltd., Guagnzhou, China), SW480 cells were incubated with RPMI-1640 medium (Sangon Biotech Co., Ltd., Guagnzhou, China) with 10% FBS, LoVo cells were incubated with F12K medium (Basal Media Co., Ltd., Shanghai, China) with 10%FBS, T84 cells were incubated with DF12 medium (Basal Media Co., Ltd., Shanghai, China) with 10%FBS, HCT-116 cells were cultured in McCoy’s 5A medium with 10% FBS. All cells were cultured at 37°C and 5% CO_2_.

### Construction of the FEZF1-AS1 knockout cell line

CRISPR/Cas9 technology was applied to construct the FEZF1-AS1 knockout colon cancer cell lines. Guide RNA (gRNA) was designed using the website of Zhang’s lab (https://zlab.bio/guide-design-resources). Three gRNA sequences targeting the first exon and one gRNA sequence targeting the last exon of FEZF1-AS1 were selected to clone into the vector spCas91.1/gRNA (Weishanglide Biotechnology Co., Ltd., Beijing, China). After validation by sequencing, the successfully incorporated vectors were transfected into HCT-116 or SW480 cells. Forty-eight hours later, medium with 3 or 2.5 μg/mL puromycin was used to screen HCT-116 or SW480 cells successfully expressing the exogenous vector, respectively. Clones were acquired by limiting dilution and verified by performing qPCR to detect the level of FEZF1-AS1. Furthermore, gene type of clones was further confirmed by PCR and sequencing, using primers flanking the gRNA sequences. The gRNA sequences for exon1 were listed in Table 2, and PCR, qPCR primers of FEZF1-AS1 were listed in Suppl. Table S1.

**Table 2:** Sequences of guide RNAs targeting FEZF1-AS1

	Sequences
^a^FEZF1-AS1_Sense guide 1	CCGATGGGCCGTCACCTCGGTTC
^a^FEZF1-AS1_Antisense guide 1	AACGAACCGAGGTGACGGCCCAT
^a^FEZF1-AS1_Sense guide 2	CCGGGGCTCGTGTCGTAGGCCAC
^a^FEZF1-AS1_Antisense guide 2	AACGTGGCCTACGACACGAGCCC
^a^FEZF1-AS1_Sense guide 3	CCGAGCCGGCGTGAACATCCACC
^a^FEZF1-AS1_Antisense guide 3	AACGGTGGATGTTCACGCCGGCT
^b^FEZF1-AS1_Sense guide 4	CCGGTCGAACCGTGTCTTGAATA
^b^FEZF1-AS1_Antisense guide 4	AACTATTCAAGACACGGTTCGAC

Note: ^a^ probes targeting the first exon. ^b^ probes targeting the last exon.

### EdU incorporation

All procedures followed the instructions of the BeyoClick EdU cell proliferation detection kit with Alexa Fluor 594 (Beyotime Biotechnology Corporation, Shanghai, China). EdU can replace thymidine and be incorporated into newly synthesized DNA in the process of DNA synthesis. On the other hand, ethynyl on EdU can react covalently with fluorescent labeled small molecule Azide Alexa Fluor 594 through the catalysis of monovalent copper ions to form a stable triazole ring, which is very rapid and called Click reaction. In brief, HCT-116 and SW480 WT and KO cells cultured in 6 well plates were incubated with EdU (10 μM). Two hours later, cells were fixed with 4% paraformaldehyde (PFA) for 15 min. Washing buffer (1 × PBS containing 3% BSA) was used to rinse the cells to completely discard the PFA. Then cells were permeated with PBS with Triton X-100 (0.3%) for 10 min followed by rinsing with washing buffer. Then cells were stained with 500 μL Azide 594 reaction solution (86% click reaction buffer, 4% CuSO_4_, 0.2% Azide 594, 10% click additive solution, volume ratio) in the dark for 30 min. Washing buffer for three times to fully remove the reaction buffer and then Hoechst 33342 was used to mark the nucleus of each cell by incubating in dark for 10 min at RT. Images were collected using a fluorescence microscope (Carl Zeiss Microscopy GmbH, Jena, Germany), The maximum excitation wavelength of Azide 594 and Hoechst 33342 is 590 and 346 nm, and the maximum emission wavelength is 615 and 460 nm.

### Colony formation

HCT-116 and SW480 cells were plated in 6 well plates at 5 × 10^3^/well and 8 × 10^3^/well, respectively. HCT-116 cells were incubated for 3 days and SW480 cells were incubated for 5 days. Before microscopic examination, the cells were fixed with 4% PFA for 15 min at room temperature and stained with 0.1% crystal violet for 10 min.

### Invasion and migration assay

Cell invasion and migration assays were performed in 24-well transwell plates with 8-μm polyethylene terephthalate membrane filters (Falcon cell culture insert; Becton-Dickinson) separating the lower and upper culture chambers. For the invasion study, Matrigel was added to the inserts of each well, while the migration assay did not need the Matrigel. HCT-116 and SW480 cells were plated on the upper chamber of the inserts at 5 × 10^4^/well and 8 × 10^4^/well respectively in serum-free medium. The bottom chamber contained medium with 10% FBS. HCT-16 cells were allowed to invasion or migrate for twenty-four hours and SW480 cells were for forty-eight hours. After the incubation period, the filter was removed, cells on the lower side of the inserts were fixed with 4% PFA for 15 min and stained with 0.1% crystal violet for 10 min. The cells on the upper side was detached using a cotton swab before counted.

### RNA pull-down plus mass spectrometry

Probes base-paired with FEZF1-AS1 were designed and labeled with biotin at the 3’ end. FEZF1-AS1 specific and negative control probes were first blocked with 0.5% BSA for 1 h and hybridized with streptavidin-coated magnetic beads for 2 h. Then the streptavidin-biotin complex was incubated with crosslinked HCT-116 or SW480 cell lysate overnight. The next day, the microbeads were washed with low salt washing buffer (100 mM Tris-HCL, 1 mM EDTA, 50 mM NaCl, 1% SDS) and high salt washing buffer (100 mM Tris-HCL, 1 mM EDTA, 300 mM NaCl) 3 times each. Finally, the microbeads were boiled for 10 min in 25 μL of 1× protein loading buffer. Differential proteins enriched by FEZF1-AS1 or control probes were picked and sent for protein mass spectrometry following SDS-PAGE and silver staining (Beyotime, Shanghai, China). Briefly, proteins were digested by a protease to form a peptide mixture, then ionized and separated by mass to charge ratio (M/Z) using a 6500 QTRAP mass spectrometer (AB SCIEX). The M/Z of each peptide could be analyzed by a mass analyzer, and the first-order mass spectrometry peak map of protein is acquired. The ion selection device automatically selects peptides with high intensity for secondary mass spectrometry analysis, outputs the secondary mass spectrometry peak map of the selected peptide. By comparing with the theoretical peak diagram, proteins are identified. Protein mass spectrometry results were validated by western blot (WB), and all probe sequences were listed in Suppl. Table S2.

### RNA Immunoprecipitation (RIP)

HCT116 cells were cross-linked with 1% formaldehyde for 10 min at 37°C, neutralized with Glycine for 5 min at 37°C and then washed with cold PBS. The cell pellet was then re-suspended in lysis buffer. Protein G microbeads (ThermoFisher, Waltham, MA, USA) were pre-hybridized in IP buffer containing 10 μg/mL yeast tRNA (TIANDZ, Beijing, China) and 10 μg/mL yeast salmon sperm DNA (Beyotime, Shanghai, China). Anti-PCK2 antibody (CST, Danvers, MA, USA) and rabbit control IgG (SAB, College Park, MD, USA) was then hybridized with protein G microbeads to form an antibody-microbead complex, which was incubated with crosslinked HCT-116 or SW480 cell lysate overnight. The next day, the microbeads were washed with low salt washing buffer and high salt washing buffer 3 times each. RNAs enriched on microbeads were eluted using elution buffer (1% SDS, 0.1 M NaHCO_3_), de-crosslinked at 55°C for 3 h, and purified using Trizol reagent (ThermoFisher, Waltham, MA, USA).

### Western blot

The cell lysate was obtained by adding cell lysis buffer (CST, Danvers MA, USA) containing proteinase inhibitor (Pierce, Waltham, MA, USA). Total proteins were separated on SDS-PAGE gel and transferred onto the PVDF membrane (GE Healthcare, Germany). After blocking with 5% non-fat milk, the protein-bound membranes were incubated with corresponding primary antibodies at 4°C overnight and HRP-coupling secondary antibodies at room temperature for 1 h. Finally, the proteins were detected using the ECL chemiluminescence instrument (Tannon, Shanghai, China). Antibodies used in this assay were listed as follows: anti-PCK2 (SAB, College Park, MD, USA), anti-PC (Proteintech, Rosemont, IL, USA), anti-MMP-2 (Servicebio, Wuhan, China), anti-c-Myc (Servicebio, Wuhan, China), anti-Bcl-2 (Servicebio, Wuhan, China), anti-β-actin (SAB, College Park, MD, USA).

### Immunofluorescence (IF)

HCT-116 and SW480 WT and KO cells were cultured on slides soaked in 6-well plates the day before. The next day, cells on the slide were fixed with 4% PFA for 15 min, washed using PBS three times, and then permeabilized with 0.5% Triton X-100 (PBS) for 20 min at room temperature. Normal goat serum (Beyotime, Shanghai, China) was next dropped on the slide and sealed at room temperature for 30 min before the required amount of diluted primary antibody was dropped on the slide. Then the slides were put in a wet box and incubated at 4°C overnight. The next day, cells on slides were incubated with the red fluorescent secondary antibody (Beyotime, Shanghai, China) at room temperature for 1 h. The nucleus of cells was re-stained using DAPI for 5 min in the dark. Lastly, the slides were sealed with sealing liquid containing an anti-fluorescence quencher, and images were collected using a fluorescence microscope.

### Targeted metabolome analysis

Cells (1 × 10^7^) were collected and re-suspended in a buffer containing acetonitrile, methanol, and deionized water (2:2:1). The sample was manipulated in Applied Protein Technology Corporation (Shanghai, China). Briefly, samples were separated using the Agilent 1290 Infinity LC ultra-performance liquid chromatography system. The mobile phase: liquid A is 10 mM Ammonium acetate aqueous solution, and B solution is acetonitrile. Samples were placed in an automatic sampler at 4°C, with a column temperature of 45°C and a flow rate of 300 μL/min. QC is set up at specific sampling intervals to evaluate the stability and repeatability of the system. The standard mixture of energy metabolites is set in the sample queue, which is used to correct the retention time in chromatography. The 5500 QTRAP mass spectrometer (AB SCIEX) was used for mass spectrometry in negative ion mode, and MRM mode was used to test the ion pair. The area and retention time of chromatographic peaks were extracted by the Multiquant software. The retention time was corrected by the standard of energy metabolites, and the metabolites were identified.

### Construction of the PCK2-overexpressing vector

The mitochondrial location vector pDsRED2-Mito (pDRM) was purchased from Waryong Biotechnology (Beijing, China). Homologous recombination was performed to clone PCK2 coding sequences into pDRM, according to the instructions of the one-step cloning kit ClonExpress II (Vazyme, Nanjing, China). Briefly, pDRM was linearized by restriction enzyme BamH I and Not I (NEB, Ipswich, MA, USA). PCK2 mRNA sequence was cloned using primers containing BamH I and Not I restriction sites and homologous sequences with the terminal sequences of linearized vector at the 5′-end (Suppl. Table S1). Then the linearized vector and the PCK2 coding sequences were ligated using ligase Exnase II supplied by the kit, and the ligation product was transfected into DH.5α. The monoclonal was picked, and pDRM-PCK2 vectors were purified using GeneJET Plasmid Midiprep Kit (Thermo Fisher, Waltham, MA, USA) and verified by sequencing.

### Construction of stable PCK2 overexpression cell lines

First, HCT-116 and SW480 cells were screened for G418, and the minimum lethal concentration for each cell line was 1.5 mg/mL and 500 μg/mL, respectively. After that, 5 × 10^5^ HCT-116 and SW480 KO cells were seeded onto 6 well plates. The next day, transfection buffer was prepared as follows: for each well, 100 μL opti-MEM medium was mixed with 0.5 μg PCK2 or non-load over-expression pDRM vectors, vortexed, and 5 μL JetPrime buffer (Polyplus, Berkeley, CA, USA) was added into the mix, vortexed again, and maintained at room temperature for 15 min. Finally, the mix buffer was added to the well containing 1 mL cell culture medium. Eight hours after transfection, cells were replaced with a fresh culture medium. Forty-eight hours later, cells were treated with a medium containing G418 for 5 days, after which the remaining cells were kept for clone selection. Clones successfully overexpressing PCK2 were cryopreserved and used for metabolome analysis and *in vivo* experiments.

### Construction of tumor-bearing mice

Methodologies of animal experiments conformed to the standards set by the Declaration of Helsinki. The study methodologies were approved by the ethics committee of the Institute of Zoology, Guangdong Academy of Sciences.

Eighteen BALB/c nude mice were bought from Guangdong Medical Lab Animal Center and randomly divided into 3 groups. Mice in each group were subcutaneously vaccinated with 1 × 10^7^ HCT-116 WT, KO, or PCK2-overexpressing cells. Five days later, the size of tumors was measured and every two days since then. Twenty days later, all mice were killed by neck-drawing, and the tumors were peeled off. After weighing, tumors undergoing paraffin embedding or protein isolation were selected by their sizes.

### Immunohistochemistry (IHC)

Paraffin-embedded tumor tissues were sliced into 4-mm sections, and the series of protein expression was determined by IHC. Briefly, after dewaxing, antigen retrieval, serum blocking, slides were incubated with anti-PCK2 (SAB, College Park, MD, USA), anti-Ki67, anti-MMP-2, anti-Bcl-2 anti-c-Myc, anti-MMP-9 (All from Servicebio, Wuhan, China), and anti-PC antibodies (Proteintech, Rosemont, IL, USA) at 4°C overnight. The subsequent steps were performed using the SABC-AP kit with anti-rabbit IgG (Beyotime, Shanghai, China). The IHC was scored as follows: the average positive intensity in the measurement area is 0, 1, 2, and 3 points. Negative without coloring (0 points). Weak yellowish (1 point). Medium-positive brownish-yellow (2 points). Positive dark brown (3 points). The positive rate of cells in the measurement area is 0, 1, 2, 3, and 4 points. 0%–15% is 0, 6%–25% is 1, 26%–50% is 2, 51%–75% is 3, and >75% is 4. The overall positive score is the positive intensity value × positive cell rate value.

### Data analysis

Survival curves were obtained with SPSS 13.0 software (SPSS Inc., Chicago, IL, USA) using Kaplan–Meier survival analysis and Log-rank test. Histograms were acquired using GraphPad Prism version 5 (Graph Pad Software, San Diego, CA, USA). Two-way ANOVA or Student’s *t*-test was performed to assess the significance of the differences. *p < 0.05 was considered statistically significant (*p < 0.05, **p < 0.01, ***p < 0.001).

## Results

### FEZF1-AS1 is upregulated in colon cancer

**Figure 1:**
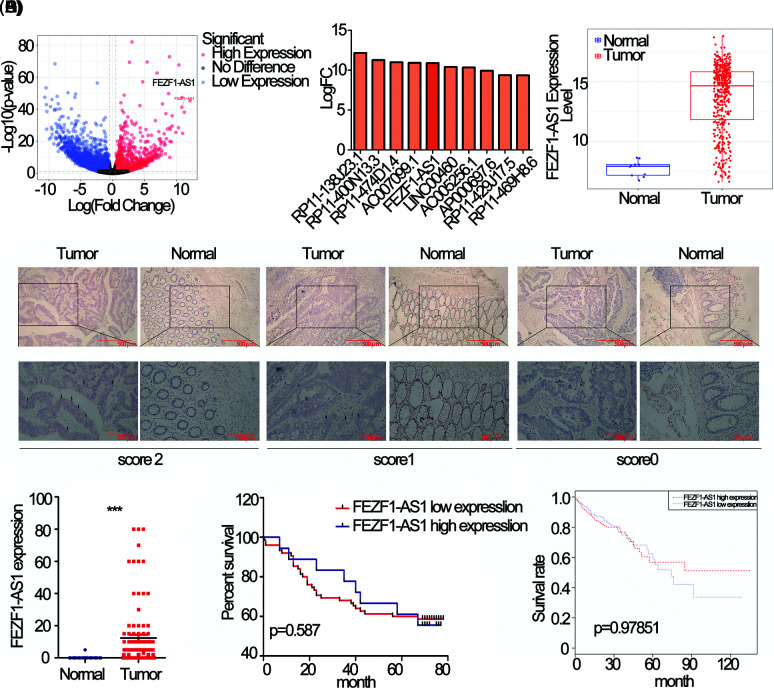
FEZF1-AS1 is upregulated in colon cancer tissues. (A) Volcano plot of all lncRNAs in colorectal cancer and normal colon tissues and data was acquired from the TCGA database. The point the arrow pointed to was FEZF1-AS1. (B) The most upregulated 10 lncRNAs in Fig. 1A. (C) Relative expression of FEZF1-AS1 in colorectal cancer and normal colon tissues and data was acquired from the TCGA database. (D) The expression of FEZF1-AS1 was detected using the RNAscope *in situ* assay in colon cancer and adjacent colon tissues. The black triangles indicate the FEZF1-AS1 blots. Scale bars, 100 μm. (E) The FEZF1-AS1 positive staining rate was quantitatively analyzed. Independent-samples *t*-test was used to analyze the significance. (F) Survival analysis of colon cancer patients overall survival based on tissue microarray chip. (G) Survival analysis of colorectal cancer patients overall survival based on TCGA database. Kaplan-Meier and log-rank was used to analyze the significance. ***p < 0.001; NS, no significance.

Differentially expressed lncRNAs in colon cancer compared to normal tissues using data deposited in TCGA was showed as volcano map, 1958 upregulated and 3953 downregulated lncRNAs respectively, standardized with twice the difference (Fig. 1A). The most upregulated 10 lncRNAs were extracted and listed separately (Fig. 1B), among which FEZF1-AS1 was chosen to be further explored. Fig. 1C showed that FEZF1-AS1 was highly expressed in colon tumors and barely expressed in normal colon, as only 10 out of 41 normal colon tissues demonstrated positive expression of FEZF1-AS1.

To confirm the upregulation of FEZF1-AS1 in colon tumor, RNAscope was performed on a colon tissue chip composed of 93 colon cancer and 83 adjacent colon tissues. Staining results indicated that FEZF1-AS1 located in the cytoplasm. All tissues were scored according to the staining intensity (Fig. 1D). Only 1 adjacent tissue was scored 1 while the others were scored 0. 15% of the colon cancer tissues were scored 2 (14 out of 93) and 48% were scored 1 (46 out of 93), while the remaining were scored 0. Relative expression of FEZF1-AS1, shown in the form of positive rates, was significantly highly expressed in tumors (Fig. 1E). However, no correlation was there between FEZF1-AS1 level with patient prognosis, either for patients from tissue chip (Fig. 1F) or patients from the TCGA database (Fig. 1G). These results confirmed that FEZF1-AS1 was significantly overexpressed in colon cancer, and barely expressed in the normal colon tissues.

### FEZF1-AS1 promotes colon cancer cell proliferation, invasion, and migration in vitro

**Figure 2:**
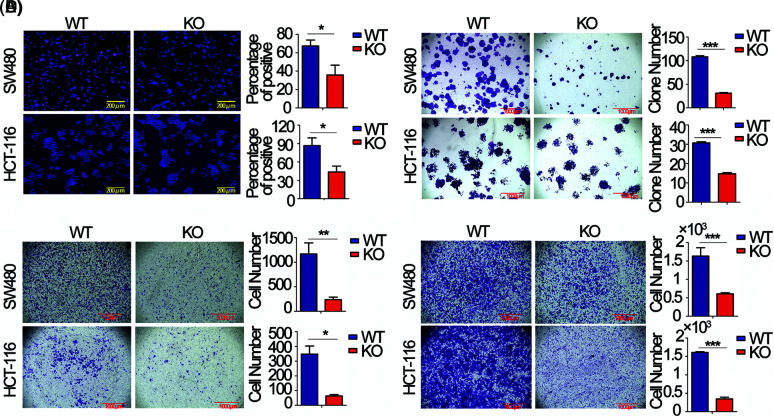
FEZF1-AS1 promotes colon cancer cell proliferation, invasion, and migration *in vitro*. (A) The effects of FEZF1-AS1 on SW480 and HCT-116 colon cancer cell proliferation were measured by EdU incorporation. The rose-red represents proliferating cells. The blue represents nuclei. Scale bars, 100 μm. Paired *t*-test, Error bars, ±SD. (B) The colony number was calculated to evaluate the effects of FEZF1-AS1 on colony formation of SW480 and HCT-116 colon cancer cells. The bars represented the average clone numbers of 15 fields, belonging to 3 independent assays. The Scale bars, 1000 μm. Paired *t*-test, Error bars, ±SEM. (C–D) Cell number was counted to evaluate the effects of FEZF1-AS1 on invasion (C) and migration (D) of SW480 and HCT-116 colon cancer cells. The bars represented the average clone numbers of 3 fields, belonging to 3 independent assays. Scale bars, 1000 μm. Paired *t*-test, Error bars, ±SEM (n = 3). *p < 0.05; **p < 0.01, ***p < 0.001.

To choose appropriate cell lines for the functional study of FEZF1-AS1. We detected the relative expression of FEZF1-AS1 in colon cancer cells LoVo, CaCo_2_, T84, HCT-116 and SW480, which were kept in our lab (Fig. S1A). Additionally, we used Depmap (Dependency Map), a valuable online tool for researchers to discover cancer vulnerabilities. This provided open access to key cancer dependency analytical and visualization tools to observe FEZF1-AS1 expression in colorectal cell lines, including 37 primary cell lines and 19 metastasis cell lines (Fig. S1B). By comprehensively considering the results above and FEZF1-AS1 expression in other reports, we chose SW480 and HCT-116 as cell models to explore mechanisms underlying FEZF1-AS1 [38]. FEZF1-AS1 knockout SW480 (SW480 KO) and HCT-116 (HCT-116 KO) cell lines were established using CRISPR/Cas9 technology (Figs. S1C–S1E) and used for functional tests. EdU incorporation assay indicated FEZF1-AS1 knockout significantly inhibited SW480 and HCT-116 cell DNA replication manifested by darker red fluorescence and fewer red positive cells (Fig. 2A). Colony formation showed fewer cell numbers of SW480 KO and HCT-116 KO cells compared with WT cells (Fig. 2B). Invasion and migration capacity was detected using transwell with or without Matrigel in the transwell insert. Figs. 2C and 2D showed fewer cells were tinted on the outside of the inserts, which were seeded with FEZF1-AS1 knockout cells. All these results demonstrated that FEZF1-AS1 promoted colon cell proliferation, invasion, and migration.

### The primary FEZF1-AS1 function in colon cancer results from being associated with phosphoenolpyruvate carboxykinase (PCK2)

**Figure 3:**
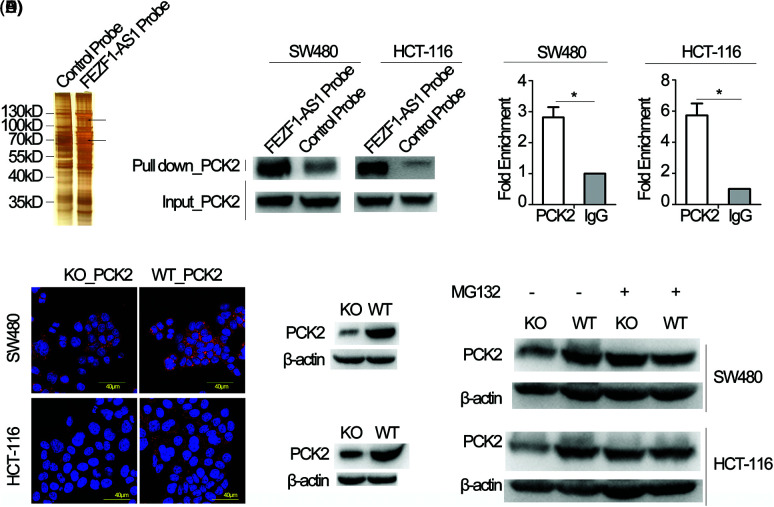
FEZF1-AS1 upregulates PCK2 protein levels by inhibiting proteasome-dependent degradation. (A) Proteins retrieved from the FEZF1-AS1 pull-down experiment after SDS-PAGE and silver staining. The strip shown by the black arrow was analyzed by mass spectrometry. (B) Western blot analysis of proteins retrieved from the FEZF1-AS1 pull-down assay using an anti-PCK2 antibody. (C) RIP assays using an anti-PCK2 antibody confirmed the enrichment of FEZF1-AS1 with PCK2. Paired *t*-test, Error bars, ±SEM (n = 3). *p < 0.05. (D) Immunofluorescence (left) and Western blot (right) assays indicated FEZF1-AS1 deficiency significantly decreased PCK2 protein level. The red fluorescence represents PCK2, and the blue fluorescence represents nuclei. Scale bars, 20 μm. (E) Protein levels of PCK2 were detected in WT or KO cells, pre-treated with MG132 (20 μmol/L) for 3 h, using western blot.

To explain the underlying mechanism of how FEZF1-AS1 exerts its oncogenic function, RNA pull-down plus protein mass spectrometry (MS) was performed to identify proteins binding to FEZF1-AS1 (Fig. 3A). All the proteins identified by MS were clustered using DAVID. According to the results of the KEGG analysis, the top five pathways are the Biosynthesis of antibiotics, the Citrate cycle (TCA cycle), Carbon metabolism, Pyruvate metabolism, and Mismatch repair, respectively (Table 3). The TCA cycle has gained increasing attention in recent years, resulting from its central role in energy metabolism. Four proteins, including PCK2 (phosphoenolpyruvate carboxykinase 2), PC (Pyruvic carboxylase), ALDOA (Fructose diphosphate aldolase A), and CS (Citrate synthase) were then chosen for further exploration. RNA pull-down plus western blot showed that only PCK2 and PC could be detected in the FEZF1-AS1 probe-enriched proteins (Figs. 3B and S2). It was reported that PCK2 regulates the TCA cycle by enhancing TCA cycle metabolite flux and functions in specific tumors [50,51]. We eventually chose PCK2 to be the subject of our research and once more verified the binding of FEZF1-AS1 and PCK2 using RIP experiments (Fig. 3C). Western blot and IF makes it clear that PCK2 was significantly downregulated in KO cells (Fig. 3D), the mechanism of which has not been reported. qPCR results showed comparable FEZF1-AS1 mRNA levels in WT and KO cells, suggesting FEZF1-AS1 regulate protein level of PCK2 independent of mRNA level (Fig. S3A). Data extracted from Depmap was used to further examine the interrelationship between FEZF1-AS1 and PCK2. Fig. S3B shows that the Pearson Correlation Coefficient of primary and metastasis cell lines was −0.131 and 0.068, respectively, and the Spearman’s rank correlation coefficient was −0.198 and 0.07, respectively. The Depmap results showed that FEZF1-AS1 level have no effect on the mRNA level of PCK2, which promoted us to speculate PCK2 was regulated at post-translational level. Ubiquitination-mediated proteasomal degradation is an important mechanism modulating protein function, and MG132 can inhibit this pathway by selectively inhibiting proteasome. Fig. 3E shows that PCK2 maintained a considerable level in KO cells with that in WT cells after treatment with MG132. This suggests FEZF1-AS1 deficiency may facilitate the binding of ubiquitin enzymes with PCK2 and promote the degradation of PCK2, the mechanism of which needs further exploration.

**Table 3:** KEGG analysis of proteins identified by MS of FEZF1-AS1 enriched proteins

Term	FDR	Fold enrichment
hsa01130:Biosynthesis of antibiotics	3.51E-04	5.624339623
hsa00020:Citrate cycle (TCA cycle)	0.001162	18.344
hsa01200:Carbon metabolism	0.007782	6.493451327
hsa00620:Pyruvate metabolism	0.026923	11.465
hsa03430:Mismatch repair	0.048353	15.95130435

### FEZF1-AS1 potentially regulates glucose utilization, TCA cycle, and OXPHOS in colon cancer cells

**Figure 4:**
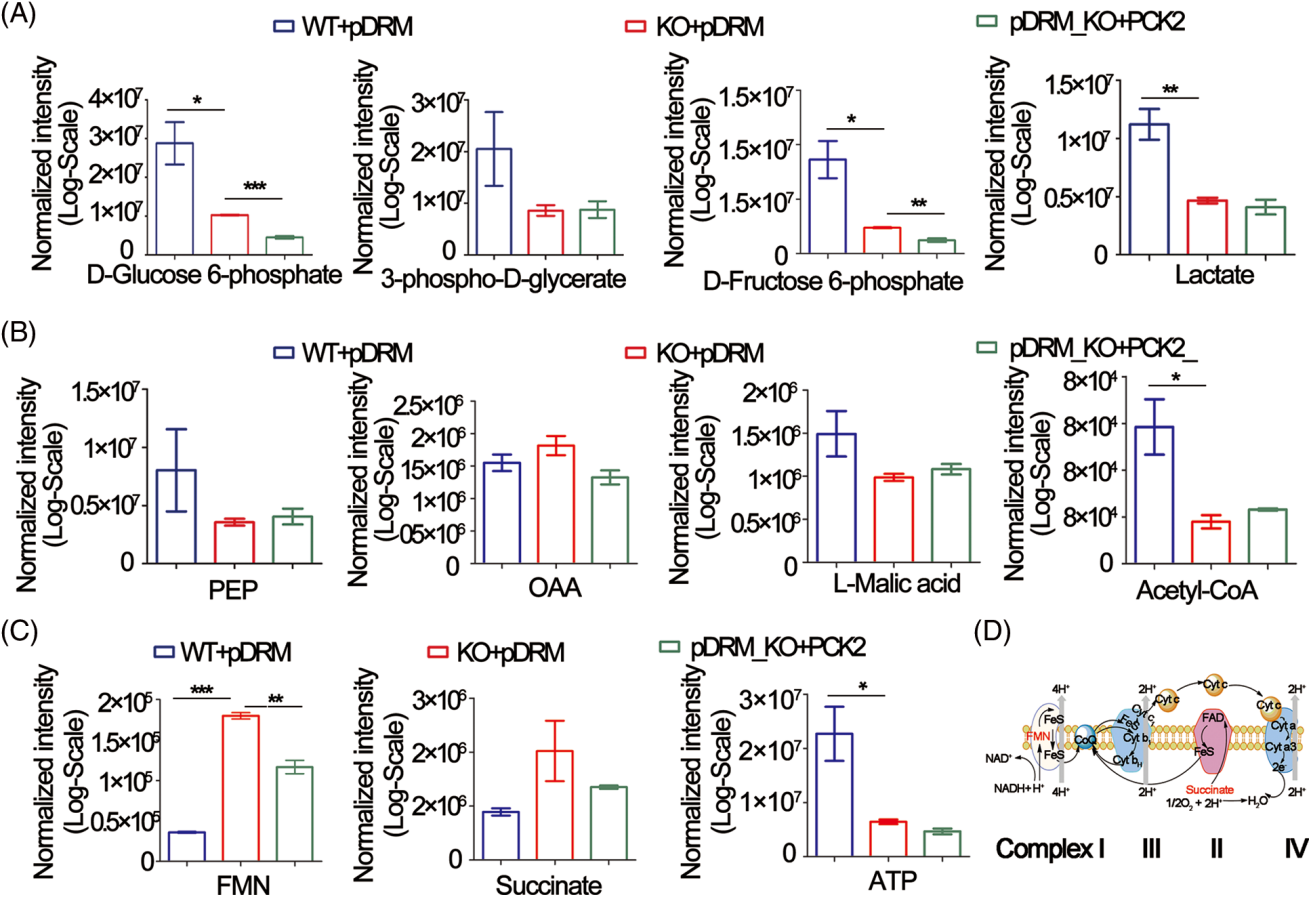
FEZF1-AS1 deficiency potentially impaired glucose utilization, TCA cycle, and OXPHOS. (A) The relative intensity of different metabolites involved in glycolysis in HCT-116 WT and KO and PCK2-overexpressing cells. (B) The relative intensity of different metabolites involved in the TCA cycle in HCT-116 WT and KO and PCK2-overexpressing cells. (C) The relative intensity of FMN and succinate involvement in OXPHOS in HCT-116 WT and KO and PCK2-overexpressing cells. (D) Sketch map of the mitochondrial respiratory chain.

PCK2 is closely related to the energy metabolism occurring in the mitochondria. We sought to determine the changes in metabolites involved in glycolysis, TCA cycle, and XOPHOS when FEZF1-AS1 was depleted. Targeted metabolomics analysis was then performed using WT or KO or PCK2-overexpressing HCT-116 cells (Fig. S4A). Fig. 4A shows that the level of D-Glucose 6-phosphate, 3-phospho-D-glycerate, D-Fructose 6-phosphate, and lactate greatly decreased when FEZF1-AS1 was decreased. In contrast, overexpression of PCK2 did not affect the level of metabolites mentioned above. This indicated that FEZF1-AS1 promotes glucose utilization in colon cancer cell HCT-116 to support tumor growth, but the effector protein downstream of FEZF1-AS1 was not PCK2. Concerning the TCA cycle, the results in Fig. 4B indicated decreased PEP level while increased OAA level in HCT-116 KO cells and PCK2 overexpression tended to reverse this effect, the phenomenon of which was consistent with the function of PCK2, that catalyzes the conversion of oxaloacetate to PEP [52]. By converting oxaloacetate to PEP, PCK2 allows for non-carbohydrate sources (glutamine, lactate, and TCA cycle intermediates) of energy to support proliferation. Consistent with this, both L-Malic acid and Acetyl-CoA of the TCA cycle were observed to first decrease and then increase in KO and PCK2-overexpressing cells, further supporting the effect of PCK2 on the TCA cycle flux (Fig. 4B). However, the significance of the metabolites participating in the TCA cycle was not realized owing to high dispersion among WT clones and the limited overexpression of PCK2. Even so, we noticed an interesting change. The level of flavin mononucleotide (FMN) and succinate was upregulated in FEZF1-AS1 knockout cells while downregulated in PCK2-overexpressing cells, especially FMN (Fig. 4C). FMN is a cofactor of the NADH-coenzyme Q oxidoreductase and succinate Q oxidoreductase, two enzymes in charge of the electronic entrance during the whole respiratory transmission chain (Fig. 4D). OXPHOS is an energy-releasing process accompanied by the production of a large amount of ATP. However, the ATP level in KO cells was not consistent with that of FMN and succinate. Impairment of the following components in the respiratory chain might result in the accumulation of FMN and succinate. All these results showed that FEZF1-AS1 deficiency potentially affected the homeostasis of energy metabolism in cell, which impeded the proliferation of colon cancer cells, and this effect was partially dependent on PCK2.

### PCK2 partly rescues FEZF1-AS1-induced impairment of colon cancer cell proliferation, invasion, and migration

**Figure 5:**
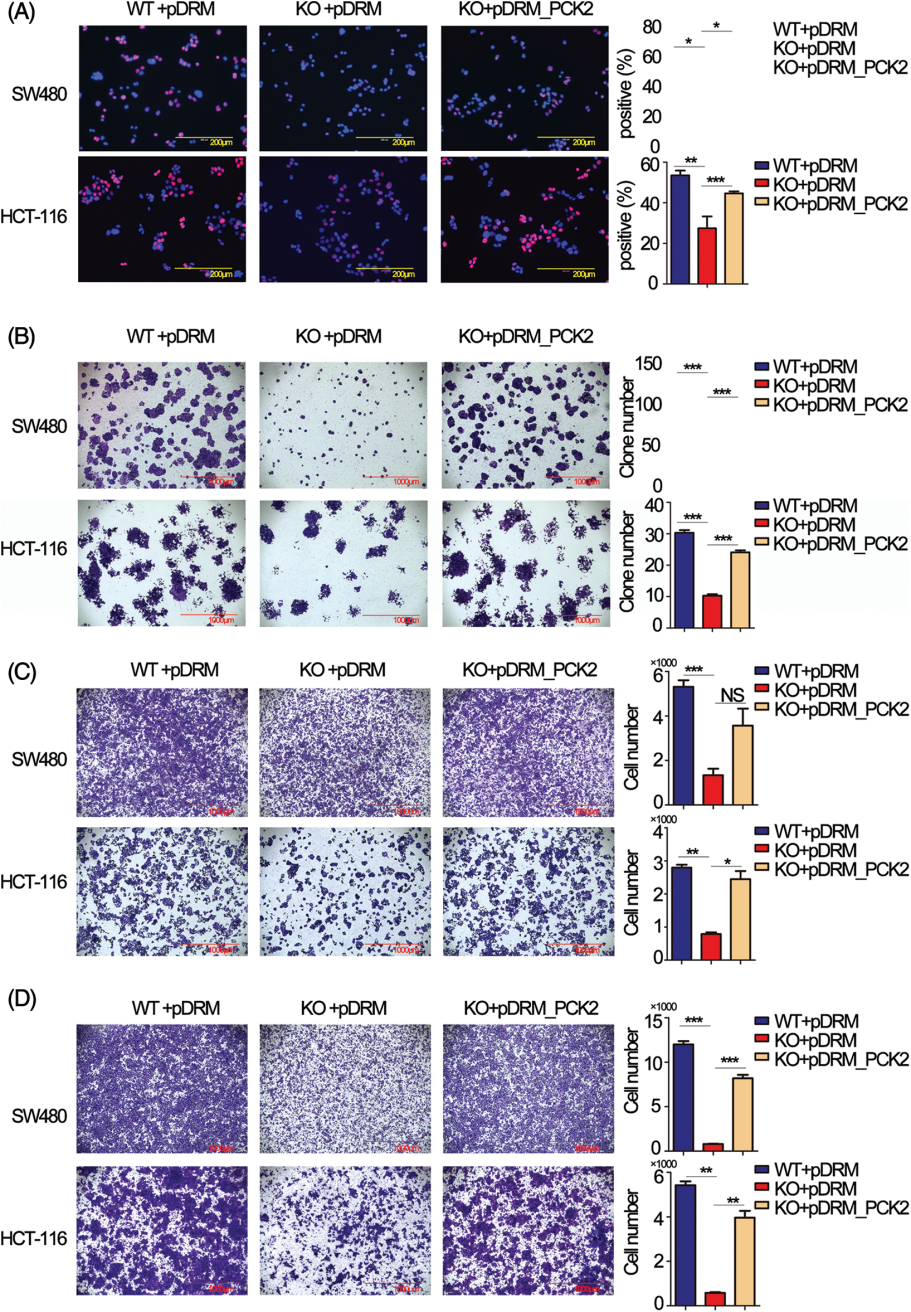
Overexpression of phosphoenolpyruvate carboxykinase (PCK2) counteracts the effect of FEZF1-AS1 deficiency *in vitro*. (A) Decreased cell proliferation (detected by EdU incorporation) in FEZF1-AS1 knockout colon cancer cells was partly rescued by PCK2 overexpression. Scale bars, 200 μm. (B) Decreased cell proliferation in FEZF1-AS1 knockout colon cancer cells was partly rescued by PCK2 overexpression, detected by colony formation. The bars represented the average clone numbers of 15 fields, belonging to 3 independent assays. Scale bars, 200 μm. Paired *t*-test, Error bars, ±SEM. (C) Decreased cell invasion in FEZF1-AS1 knockout colon cancer cells was partly rescued by PCK2 overexpression, detected by transwell invasion. The bars represented the average clone numbers of 3 fields, belonging to 3 independent assays. Scale bars, 200 μm. Paired *t*-test, Error bars, ±SEM. (D) Decreased cell migration in FEZF1-AS1 knockout colon cancer cells was partly rescued by PCK2 overexpression. The bars represented the average clone numbers of 3 fields, belonging to 3 independent assays. Scale bars, 200 μm. Paired *t*-test, Error bars, ±SEM (n = 3). *p < 0.05, **p < 0.01, ***p < 0.001.

Rescue experiments were performed to further confirm that PCK2 was the downstream executor of FEZF1-AS1. The coding sequence of PCK2 was cloned to the mitochondrial localization vector pDsRED2-Mito (pDRM), and the expression was verified by IF and western blot (Figs. S4B and S4C). EdU incorporation results showed increased PCK2 levels in KO cells remarkably enhanced the number of proliferating cells (Fig. 5A). Colony formation further confirmed the proliferation-promoting effect of PCK2 on SW480 and HCT-116 cells (Fig. 5B). Coincidentally, overexpression of PCK2 in KO cells counteracted the inhibitory effect of FEZF1-AS1 on cell invasion and migration and remarkably increased the cell numbers on the outside of the inserts (Figs. 5C and 5D). These results suggested that PCK2 overexpression, if not all, at least partially rescued the proliferation, invasion, and migration capacity of KO cells.

### FEZF1-AS1 promotes colon cancer progression in vivo

**Figure 6:**
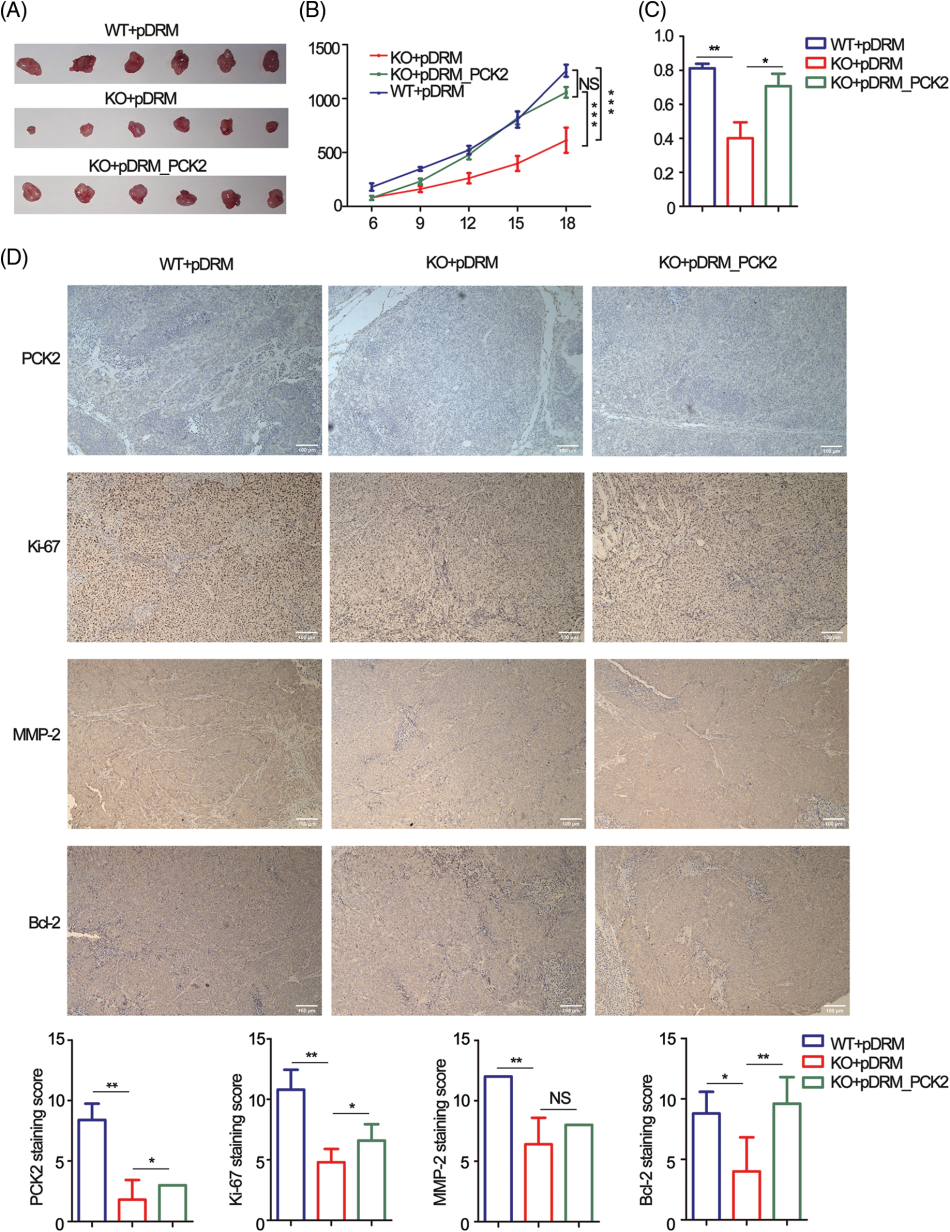
PCK2 partly rescued the proliferation inhibitory effect of FEZF1-AS1 deficiency *in vivo*. (A) The effect of FEZF1-AS1 and PCK2 on tumor proliferation in a nude mouse xenograft model. Images of tumors from the HCT-116 WT or KO or PCK2-overexpressing groups (*n* = 6 for each group). (B) Six days after subcutaneous vaccination, tumor sizes were measured every two days and calculated by length × width^2^/2. Two-way ANOVA was used for analysis (*n* = 6 for each group) (C) A bar graph of tumor weight from Fig. 6A. (*n* = 6 for each group) (D) Representative images of immunohistochemistry staining of PCK2, Ki-67, MMP-2, and Bcl-2 in tumors from Fig. 6A (above) and the positive staining sores of each protein were listed below. Scale bars, 100 μm. Independent *t*-test, Error bars, ±SEM. *p < 0.05; **p < 0.01, ***p < 0.001.

Tumorbearing BALB/c nude mice were constructed to confirm the oncogenic role of FEZF1-AS1 *in vivo*. HCT-116 WT or KO or PCK2-overexpressing cells were subcutaneously injected into nude mice. Three weeks later, all mice were sacrificed. Exfoliated tumors (Fig. 6A) indicated that the tumorigenic ability of FEZF1-AS1 deficient HCT-116 cells was greatly impaired, which could be rescued by PCK2 overexpression. Statistically, tumor sizes induced by HCT-116 KO cells were almost a quarter of those induced by HCT-116 WT cells, while PCK2 notably promoted tumor growth of KO cells (Fig. 6B). A similar result was obtained after all tumors were weighed (Fig. 6C). To further understand the characteristics of these tumor tissues, we performed IHC experiments. IHC of PCK2 confirmed that FEZF1-AS1 improves PCK2 protein level (Fig. 6D). Positive staining of Ki-67, c-Myc, MMP-2, and Bcl-2 was significantly lower in HCT-116 KO cell-induced tumors and upregulated after PCK2 overexpression, suggesting that the proliferation and invasion capacity was regained (Figs. 6D and S5A). In addition, proteins extracted from tumors were detected by WB using the same primary antibody. The results indicated that the protein levels of PC, PCK2, MMP-2, and Bcl-2 were much lower in KO cells induced tumors (Fig. S5B). All these data showed that FEZF1-AS1 promoted colon cancer cell proliferation *in vivo*.

## Discussion

LncRNAs have long been reported to be involved in cancer development. However, who is the most important, does the most upregulated means the most important? Considering this, we screened all the dysregulated lncRNAs in colon cancer tissues acquired from the TCGA database and picked FEZF1-AS1 for further exploration. FEZF1-AS1 is among the top ten upregulated lncRNAs in colon cancer tissues, and this was confirmed by RNAscope *in situ* hybridization performed on colon cancer chips and was consistent with previous conclusions [53,54]. Functional experiments using FEZF1-AS1 knockout colon cancer cells showed FEZF1-AS1 significantly promoted colon cancer cell proliferation *in vitro* and *in vivo*. Notably, FEZF1-AS1 showed tumor-promoting ability in other tumors like gastric tumors, non-small cell lung cancer, and pancreatic ductal adenocarcinoma (PDAC), which indicated that FEZF1-AS1 might serve as an index in tumor detection [18,19,55]. Contrary to our predictions, the expression of FEZF1-AS1 does not correlate with patient prognosis, and the survival analysis from TCGA also shows no correlation between FEZF1-AS1 expression and patients’ survival. There is another report indicating that FEZF1-AS1 is an independent prognostic factor for colorectal cancer [53]. The discrepancies in these outcomes may result from the different cohorts of patients and methodologies used in the analyses.

The RNA pull-down assay and mass spectrometry identified a series of mitochondrial proteins that potentially interacted with FEZF1-AS1, among which PCK2 was finally confirmed to be the primary one using WB and RIP. Metabolic reprogramming is a typical strategy for a tumor cell to support proliferation and metastasis [55–58]. Aerobic glycolysis (the Warburg effect) has received the most attention and has been extensively reviewed [59–61]. In PDAC, FEZF1-AS1 silencing was proved to inhibit glucose uptake and lactate production by absorbing miR-107, which targets ZNF312B [19]. In this study, PCK2 protein level decreased significantly in SW480 and HCT-116 KO cells. Subsequently, targeted metabolome analysis was performed in FEZF1-AS1 knockout HCT-116 and PCK2-overexpressing HCT-116 cells. As expected, intermediates in glycolysis like D-Glucose 6-phosphate and Lactate decreased significantly in KO cells and could not be rescued by PCK2 overexpression, suggesting that other proteins interacted with FEZF1-AS1 to regulate the utilization of glucose in colon cancer cells. PKM2 maybe one of the proteins. Bian et al. [53] revealed that FEZF1-AS1 increases colorectal cancer cell proliferation and metastasis by regulating the PKM2/STAT3 signaling pathway and glycolysis. However, we did not find the PKM2 peptide in our mass spectrometry results. This may be because of the different cell culture and protein detection methods.

Moreover, we found that the PEP level was decreased, and OAA was slightly upregulated in KO cells, together with other metabolites in the TCA cycle like L-Malic acid and Acetyl-CoA. PCK2 overexpression tends to counteract the effect of FEZF1-AS1. The TCA cycle is composed of a series of biochemical reactions occurring in the mitochondrial matrix, oxidizing fuel sources to provide energy and macromolecules to the cell and maintain the redox balance [27]. While glucose provides the main source of pyruvate entering the TCA cycle in normal cells, cancer cells tend to replenish the TCA cycle through glutaminolysis [62]. FEZF1-AS1 induced a decrease of PCK2-depleted TCA intermediates and inhibited colon cancer cell proliferation. However, more clones were needed to confirm the results, and carbon-tracing experiments needed to be done to confirm if PCK2 affected glutaminolysis.

The most interesting finding is the upregulation of FMN and succinate in KO cells and the following downregulation after PCK2 overexpression. There has been one report that elevated cecal succinate may inhibit colon cancer cell proliferation and angiogenesis [63]. However, the mechanisms underlying this were not uncovered. We speculate that FEZF1-AS1 is associated with enzymes in complex III and IV on the respiratory chain, inhibits the transmission of electrons, and finally induces the accumulation of FMN and succinate. However, more experiments are needed to explore this mechanism further.

In addition to metabolism regulation, FEZF1-AS1 was reported to promote gastric cancer cell proliferation by binding with lysine-specific demethylase 1 (LSD1) to epigenetically repress the expression of p21 [15]. In colon cancer, FEZF1-AS1 was mainly located in the cytoplasm, and we did not consider proteins located in the nucleus. In addition to PCK2, we also noticed another protein, pyruvic carboxylase (PC), to be regulated by FEZF1-AS1 (Figs. S2B and S6). PC catalyzes the first step of gluconeogenesis, upstream of PCK2, and plays a vital role in regulating TCA cycles [64,65]. However, how FEZF1-AS1 regulated these proteins to reprogram cell metabolism needs to be investigated.

PCK2 activity is greatly dependent on the level of mitochondrial GTP. Other regulatory factors, such as ATF4, or HIF, bind to the promoter of PCK2 to enhance its transcription in breast and cervix carcinomas or melanoma, respectively [46,47]. In this article, FEZF1-AS1 was found to increase the protein, but not mRNA level of PCK2, which indicated a post-translational regulatory mechanism. Previous reports showed that FEZF1-AS1 promoted the stability of PKM2 by reducing ubiquitination mediated protein degradation. Similar mechanism was applicable for lncRNA GCASPC, downregulating PC protein abundance via the ubiquitination-proteasome pathway in gallbladder cancer [53,66]. However, we did not reveal whether FEZF1-AS1 interacted with PCK2 directly or indirectly and how FEZF1-AS1 regulates PCK2 protein levels. Further exploration needs to be done to uncover the underlying mechanisms. Briefly, we showed that the lncRNA FEZF1-AS1 was up-regulated in colon cancer tissues and functioned as oncogene by interacting with PCK2 to regulate cellular metabolism.

## Data Availability

Supplemental Figures or Tables are available at https://entuedu-my.sharepoint.com/:b:/g/personal/huamin_wang_staff_main_ntu_edu_sg/Eens9iM2N3lEjpaptjri_T0BT2zVN_j-1kL88QfUcSRCrA?e=3YbxOd. Datasets used and/or analyzed in this study are available from the corresponding author on reasonable request.
